# Household satisfaction and associated factors with community-based health insurance scheme in Ethiopia: systematic review and meta-analysis

**DOI:** 10.1186/s41256-023-00325-y

**Published:** 2023-09-15

**Authors:** Daniel Tarekegn Worede, Mengistie Kassahun Tariku, Melash Belachew Asresie, Belayneh Fentahun Shibesh

**Affiliations:** 1 Department of Public Health, College of Medicine and Health Science, Debre Markos University, 269, Debre Markos, Ethiopia; 2 Department of Epidemiology and Biostatistics, School of Public Health, College of Medicine and Health Science, Bahir Dar University, 79, Bahir Dar, Ethiopia; 3 Department of Reproductive and Population Health School of Public Health, College of Medicine and Health Science, Bahir Dar University, 79, Bahir Dar, Ethiopia

**Keywords:** Household satisfaction, Associated factors, Health insurance, Ethiopia, Systematic review and meta-analysis

## Abstract

**Background:**

Community-based health insurance (CBHI) schemes are crucial for households to avoid financial hardship, improve healthcare quality, and engage in health policies. Household satisfaction is a key indicator for assessing healthcare quality and identifying service gaps. However, research on household satisfaction with CBHI in Ethiopia is limited. Therefore, this study aimed to evaluate household satisfaction and associated factors with CBHI schemes in Ethiopia.

**Methods:**

A comprehensive search of relevant literature was conducted using multiple databases, including PubMed, Google Scholar, Africa Journal Online, and Ethiopian Universities' institutional open-access online repositories. The search was carried out between January 25, 2023, and February 28, 2023. Twelve primary studies, including eight published and four unpublished, were identified and included in the analysis with a total sample size of 5311 participants. A protocol with the registration number CRD20531345698 is recorded on the Prospero database. Two authors, DT and MK, independently extracted the required data using a standardized form. The extracted data were then analyzed using STATA version 17 software. Heterogeneity was assessed using the Cochrane Q-test and I^2^ tests. Finally, a random-effect model was employed to calculate the overall household satisfaction with CBHI and to determine the associated factors.

**Results:**

The meta-analysis showed that the overall household satisfaction with CBHI in Ethiopia was 62.26% (95% CI 53.25–71.21%). The study found regional variations in household satisfaction, with 63.40% in Oromia, 64.01% in Amhara, 49.58% in Addis Ababa, and 66.76% in SNNPs. The study identified several factors associated with household satisfaction and the CBHI scheme, including the availability of drugs (OR 2.13, 95% CI 1.47–2.78), friendly services (OR 3.85, 95% CI 1.60–6.10), affordability of premium (OR 2.80, 95% CI 1.97–3.63), and knowledge/awareness of CBHI (OR 2.52, 95% CI 1.73–3.33).

**Conclusions:**

The study provides valuable insights into household satisfaction with CBHI in Ethiopia, with a considerable proportion of enrolees being satisfied. The finding highlights regional variations in household satisfaction and underscores the need for tailored interventions and monitoring to enhance CBHI sustainability and effectiveness. The results suggest that healthcare providers and policymakers should prioritize the availability of drugs, friendly services, affordable premiums, and education to improve household satisfaction with CBHI schemes.

## Background

According to a ‘leave-no-one behind’ global commitment, countries worldwide are trying to achieve universal health coverage (UHC). However, financing quality healthcare is a big challenge in low-income countries [1]. In emerging nations, numerous individuals are pushed into dire poverty due to the significant expenses related to out-of-pocket (OOP) catastrophic healthcare payments, making it challenging to obtain quality healthcare services [2]. Policymakers established an innovative health insurance scheme called community-based health insurance (CBHI) to protect poor people from financial hardship and falling into poverty [3]. Community-based health insurance is a microfinance mechanism among rural communities as a volunteer-based health insurance strategy to share risks among households and pool money to finance healthcare services, leading to universal health coverage [4].

CBHI aims to foster inclusiveness and ensure individuals' access to essential healthcare services, grounded in the values of solidarity and reciprocal support among participants who join of their own accord. This approach even encompasses the possibility of excluding low-income households from healthcare usage if they are unable to cover the fees due to their financial situation [5, 6]. However, implementing CBHI schemes in lower- and middle-income countries (LMICs) has faced significant challenges, particularly in terms of low levels of participation and variations across different communities and regions. These persistent issues have resulted in continued difficulties for many communities accessing the healthcare system, primarily due to financial constraints.

Evidence showed that various sociodemographic characteristics, including age, educational status, income, and distance to healthcare facilities, influence enrolment in CBHI schemes. Additionally, factors related to the health facilities, such as the quality of care provided, trust in administrators, and access to information about CBHI, also play crucial roles in determining CBHI membership [7, 8]. Another challenge LMICs face in the context of CBHI schemes is a high dropout rate among enrolled members [9, 10]. It ranges from 46 to 80% in India [11], 35% in Ghana [12], 46% in Burkina Faso [13], and 58–83% in Senegal [14]. Client dissatisfaction has been consistently reported as a significant factor contributing to these dropout rates [15, 16]. Specifically, dissatisfaction is often linked to concerns about the quality of care received [11, 17, 18], unavailability of prescribed medications at contracted health facilities [19, 20], misbehavior of health professionals [21], and the distance individuals must travel to reach the health facility [19, 20], are prompting factors reported to discontinue their participation in CBHI schemes.

In Ethiopia, where 80% of the population lives in rural parts, it is challenging to finance healthcare to access quality health services to all [22]. To overcome these financial challenges and achieve universal health coverage in rural communities, the Ethiopian government launched the CBHI scheme in 2011 [23]. This pilot program was rolled out in 13 districts of four regions: Tigray, Amhara, Oromia, and South Nation Nationalities and Peoples (SNNPs). The scheme then expanded to 700 districts and cities, reaching 32.2 million people in 2019/2020 [24]. Since CBHI is a new health policy in the country, robust evidence is crucial to meet its implementation goal. The latest study in Ethiopia by Yibeltal assessing the impact of community-based health insurance on health service utilization and financial risk protection found that CBHI membership increases health services utilization and financial risk protection. It proves an essential strategy to promote universal health coverage in Ethiopia [25].

The CBHI dropout rate in Ethiopia is far too high, ranging from 18 to 37% [20, 26]. Evidence showed that dissatisfaction with healthcare services and constraints to paying a premium are barriers to enrolment and membership renewal [24]. Client satisfaction is the customer**'**s judgment on the quality and outcome of care. It is the degree of satisfaction with the process and product of care and the extent that the customer feels their needs and expectations are met. It is one of the attributes of quality healthcare service outcomes and one of the key performance indicators to assess the quality of healthcare delivery and measure the effectiveness of healthcare services [27].

One of healthcare providers' primary objectives is meeting patients' expectations. Client satisfaction has become the benchmark for evaluating the performance of the healthcare system because CBHI enrolees expect a better quality of care. CBHI enrolees satisfied with the healthcare services provided with this scheme are more likely to continue using services and renew a membership than dissatisfied members [28]. Studies also identified that health services-related factors affect CBHI enrolees' satisfaction with health services under the CBHI scheme in addition to demographic and socioeconomic factors [29]. Still, more robust evidence of enrolees' satisfaction with the CBHI scheme is crucial in Ethiopia to improve healthcare services, amending insurance policies, and providing feedback on the quality and availability of healthcare services.

There are some studies about household and individual satisfaction from each corner of the country, but no single study shows household and individual satisfaction with the CBHI at a national level. Understanding community satisfaction with CBHI is essential for decision-makers to take action to steer implementation. Systematic reviews and meta-analyses provide the best and most robust available evidence estimates of household satisfaction with the CBHI scheme nationally.

Therefore, this systematic review and meta-analysis aimed to investigate household satisfaction and associated factors with the CBHI scheme in Ethiopia.

## Methods

### Study design and setting

Systematic review and meta-analysis study design was used to determine pooled household satisfaction and associated factors with community-based health insurance in Ethiopia. This meta-analysis includes primary studies that assessed household or client satisfaction and associated factors within Ethiopia's CBHI scheme. Ethiopia is the second most populous country, next to Nigeria in Africa. It is the hub of about 126.5 million inhabitants in the lower-income economic status in the eastern African region [30]. Financing quality healthcare services for its entire people is challenging for a government.

To avoid duplication of work, we checked the title to determine whether a systematic review and meta-analysis were already conducted or not using the trial registration number and Cochrane database. Preferred reporting items for systematic review and meta-analysis (PRISMA) 2020 explanation and elaboration: updated guidance and exemplars for reporting systematic reviews protocol were followed for reviewing the literature [31]. A meta-analysis is registered on the Prospective International Register of Systematic Reviews (PROSPERO) database with the registration number CRD20531345698. We searched major databases, including PubMed, Google Scholar, Africa Journal Online, Addis Ababa University, Bahir Dar University, and Jimma University's institutional open-access online repositories were used to identify all relevant literature. EndNote version 8 citation tools to facilitate review and citation are applied. We extended our search to retrieve additional literature using the references list of identified studies. Furthermore, unpublished literature from Ethiopia Universities' online database is accessed.

Population, exposure, comparison, and outcome (PECO) were applied to the frame and answered systematic review and meta-analysis questions. Population: household head, exposure: a determinant of household satisfaction, comparator: reported reference group in each included study, and outcome: level of household satisfaction.

A literature search was conducted between January 25, 2023, and February 28, 2023, using the following terms: "household" OR "client" AND "satisfaction" OR "associated factors" OR "community-based health insurance" AND "Ethiopia," combined with boolean operators.

### Eligibility criteria

All observational studies with defined household or client satisfaction outcomes with community-based health insurance reported in English were eligible and included in a meta-analysis. Furthermore, the inclusion also considered all published and unpublished studies in Ethiopia. Meanwhile, we excluded studies in a meta-analysis with methodological problems, not fully accessible, and undefined outcomes, letters, reviews, commentary, and studies done outside Ethiopia.

### Measurement of outcome variables

A meta-analysis has two outcomes. The primary outcome variable is household satisfaction, measured as the number of households satisfied with healthcare services under the CBHI scheme divided by the total study size multiplied by 100. The second outcome variable is associated factors to household satisfaction, measured using odds ratio (OR) and calculated based on the binary result from primary studies included in the analysis.

### Quality assessment and data abstraction

Two authors (DT and MK) screened titles and abstracts independently. Those two authors conducted full-length article reviews for inclusion and exclusion, quality appraisal, and data collection for systematic review and meta-analysis. Then all studies identified via databases and grey literature were subjected to full-text assessment. In this meta-analysis, in cases of disagreements among the researchers, we arranged to meet with the third author (MB) to facilitate discussion and arrive at a consensus decision. This allowed us to carefully consider all perspectives and arrive at a conclusion that was agreed upon by the entire team. It is important to note that such collaboration and open communication is essential in conducting rigorous and accurate meta-analyses. The quality of each study was appraised using the Joanna Briggs Institute (JBI) quality check tool for observational studies [32]. This quality check tool has eight-item checklists to assess the quality of studies, including 1. assessing inclusion and exclusion criteria, 2. description of study subject and setting, 3. measurement of outcome, 4. measurement of exposure, 5. identification of confounding factors, 6. approaches for controlling confounders, 7. appropriate statistical analysis, and 8. objective and standard criteria used. We collected study details, including the first author's name, year of publication, study design, region (location), sample size, the proportion of practice and awareness separately, and study outcome from primary studies. Twelve studies with ≥ 6 out of 8 scales were considered acceptable quality for this systematic review and meta-analysis. All necessary data items were collected using a standardized data extraction Excel form (Table 1).

**Table 1:** Summary of studies included for systematic review and meta-analysis to estimate overall household satisfaction with community-based health insurance in Ethiopia

ID	Author	Year of publication	Region	Design	Setting	Participants	N	(P)%	RR
1	Badacho et al. [24]	2016	SNNPs	CS	Community	HH head	386	91.38	100
2	Abera et al. [34]	2017	Amhara	CS	Institution	Patients	612	77.6	98
3	Addis et al. [35]	2021	SNNPs	CS	Community	HH head	627	54.1	100
4	Dawit [37]	2020	AA	CS	community	HH head	366	53	100
5	Mitiku and Geberetsadik [38]	2019	SNNPs	CS	Community	HH head	528	54.7	100
6	Alelign [39]	2021	Amhara	CS	Institution	Patients	393	63	100
7	Yasab [40]	2021	Amhara	CS	Community	HH head	604	56.1	100
8	Kalkidan [41]	2021	Amhara	CS	Community	HH head	346	77.6	100
9	Fufa [42]	2021	Oromia	CS	Institution	Patients	399	63.4	100
10	Esubalew [43]	2023	Amhara	CS	Institution	Patients	314	59	100
11	Biruktawit [44]	2021	AA	CS	Institution	Patients	419	46.3	96.4
12	Aragaw [45]	2019	Amhara	CS	Institution	Patients	317	50.2	100

*N* sample size, *RR* response rate, *CS* cross-sectional, *HH* household head, *AA* Addis Ababa, *p* prevalence

### Data processing and analysis

For citation, screened and eligible articles were entered into Endnote version 8, and extracted data was imported to STATA 17 statistical software for analysis. Q-test and inverse variance (I^2^) statistical tests on forest plots were used to assess the presence and degree of heterogeneity among the studies, respectively. A *p* value ≤ 0.05 was considered a statistically significant heterogeneity. A heterogeneity test (I^2^) results for studies were considered 0%, 25%, 50%, and 75% as no, low, moderate, and high degrees of heterogeneity, respectively [33]. Leave-one-out sensitivity analyses were done to determine the source of heterogeneity among studies. The pooled values of household satisfaction levels were estimated using the random effects meta-analysis model, generating the pooled 95% CI using Der Simonian and Laird's methods. Sub-group analysis was done based on location to minimize the random variations between the point estimates of the primary studies. Univariate meta-regression was done to identify the possible source of heterogeneity, and meta-regression was computed to identify the associated factors with practice. Publication bias was checked using Egger's tests at a 5% significant level. Point estimate and 95% confidence intervals were displayed in the forest plot format in this plot; the size of each box indicated the weight of the study, while each crossed line refers to 95% confidence intervals.

## Results

### Study selection

**Fig. 1:**
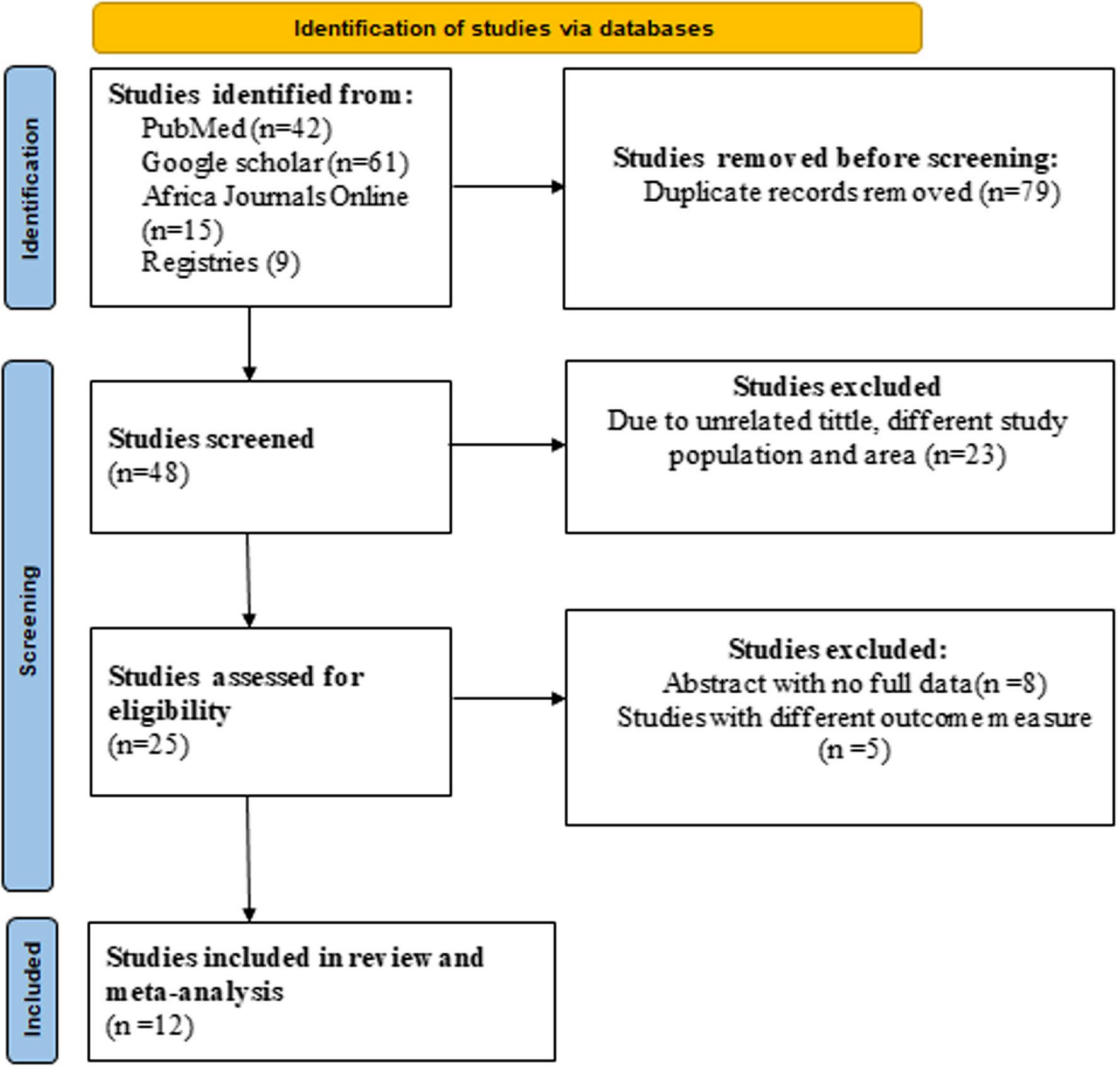
A flow diagram depicting studies included in a systematic review and a meta-analysis to estimate the pooled household satisfaction with community-based health insurance in Ethiopia

Initially, 127 primary studies were identified through various databases and registry searches. After excluding 79 studies due to duplication, 23 studies were further eliminated based on titles and abstracts that were unrelated to the research topic. The remaining 25 full-text articles underwent a rigorous screening process to assess their eligibility for inclusion in the meta-analysis. Of the 25 full-text articles, 13 studies were excluded because they did not meet the eligibility criteria. The criteria for exclusion included studies that did not focus on household or individual client satisfaction, methodological problems, inaccessible or undefined results, letters, reviews, commentary, and studies conducted outside of Ethiopia. Finally, 12 primary studies [24, 34–44] that scored six or higher out of eight on the JBI quality score were considered suitable for systematic review and meta-analysis. These studies focused on household or individual client satisfaction in Ethiopia and had high-quality research methodologies. Therefore, the selection process was extensive and rigorous to ensure that the studies included in the meta-analysis were of high quality and provided relevant information on the topic of interest (Fig. 1).

### Study characteristics

A meta-analysis was conducted to examine 12 primary studies that were carried out in Ethiopia. These studies were conducted in the three largest regions of the country and the Addis Ababa city administration. The studies included six from Amhara [34, 35, 39, 40, 43, 45], three from SNNPs [24, 35, 38], two from Addis Ababa [36, 37], and one from Oromia [42]. The sample sizes of the studies varied from 314 to 627, and the total study size was 5311 (as shown in Table 1). All the studies were cross-sectional observational epidemiological studies published between 2016 and 2023. Two of the 12 studies [42, 44] employed mixed methods using both qualitative and quantitative data collection techniques. These studies were conducted among households that purposely selected CBHI enrollees**.**

### Results of study bias assessment

Each paper underwent a thorough assessment, classifying studies as either low risk or good quality if they scored 8 out of 8, while studies scoring 6 or 7 were deemed medium risk. No study was excluded from the reviews using the above appraisal tools. The following criteria were utilized to evaluate cross-sectional studies: (1) adherence to inclusion criteria, (2) clear description of study subjects and settings, (3) utilization of valid and reliable exposure measurements, (4) utilization of objective and standardized criteria, (5) identification of confounding factors, (6) implementation of strategies to address confounding, (7) appropriate outcome measurement, and (8) utilization of suitable statistical analysis (Table 2).

**Table 2:** Quality appraisal results of included cross-sectional studies in Ethiopia, Using Joanna Briggs Institute (JBI) quality appraisal checklist for systematic review and meta-analysis

Included articles	Criterion number								Score	Risk
	1	2	3	4	5	6	7	8		
Badacho et al. [24]	✓	✓	✓	✓	✓	✓	✓	✓	8/8	Low
Abera et al. [34]	✓	✓	✓	✓	✓	✓	✓	✓	8/8	Low
Addis et al. [35]	✓	✓	✓	✓	✓	✓	✓	✓	8/8	Low
Gashaw [37]	✓	✓	✓	✓	✓	✓	✓	✓	8/8	Low
Mitiku and Geberetsadik [38]	✓	✓	✓	✓	✓	✓	✓	✓	8/8	Low
Alelign and Eniyew [39]	✓	✓	✓	✓	✓	✓	X	✓	7/8	Medium
Yasab [40]	✓	✓	✓	✓	X	X	✓	✓	7/8	Medium
Kalkidan [41]	✓	✓	✓	✓	✓	✓	✓	✓	8/8	Low
Fufa et al. [42]	✓	✓	✓	✓	✓	✓	✓	✓	8/8	Low
Esubalew [43]	✓	✓	✓	X	✓	✓	✓	X	6/8	Medium
Biruktawit [44]	✓	✓	✓	X	✓	✓	X	✓	6/8	Medium
Abebe [45]	✓	✓	✓	✓	X	X	✓	✓	6/8	Medium

### Household satisfaction with community-based health insurance in Ethiopia

This study conducted a meta-analysis and systematic review to evaluate household satisfaction with community-based health insurance (CBHI) in Ethiopia. The research gathered data from various sources and calculated household satisfaction by dividing the number of satisfied household heads by the total study size and then multiplying the results by 100.

The meta-analysis findings showed that the overall household satisfaction with CBHI in Ethiopia was 62.26%, with a 95% confidence interval ranging from 53.25 to 71.21%. These results suggest that many households in Ethiopia are satisfied with CBHI. The study also used a forest plot to visualize the meta-analysis results.

A visualized forest plot revealed that the SNNP region had the highest household satisfaction reported by Badacho et al. [24]. The second-highest level of satisfaction was reported in the Amhara region by Abera Af et al. [34]. These results indicate that there are regional variations in the level of household satisfaction with CBHI in Ethiopia.

**Fig. 2:**
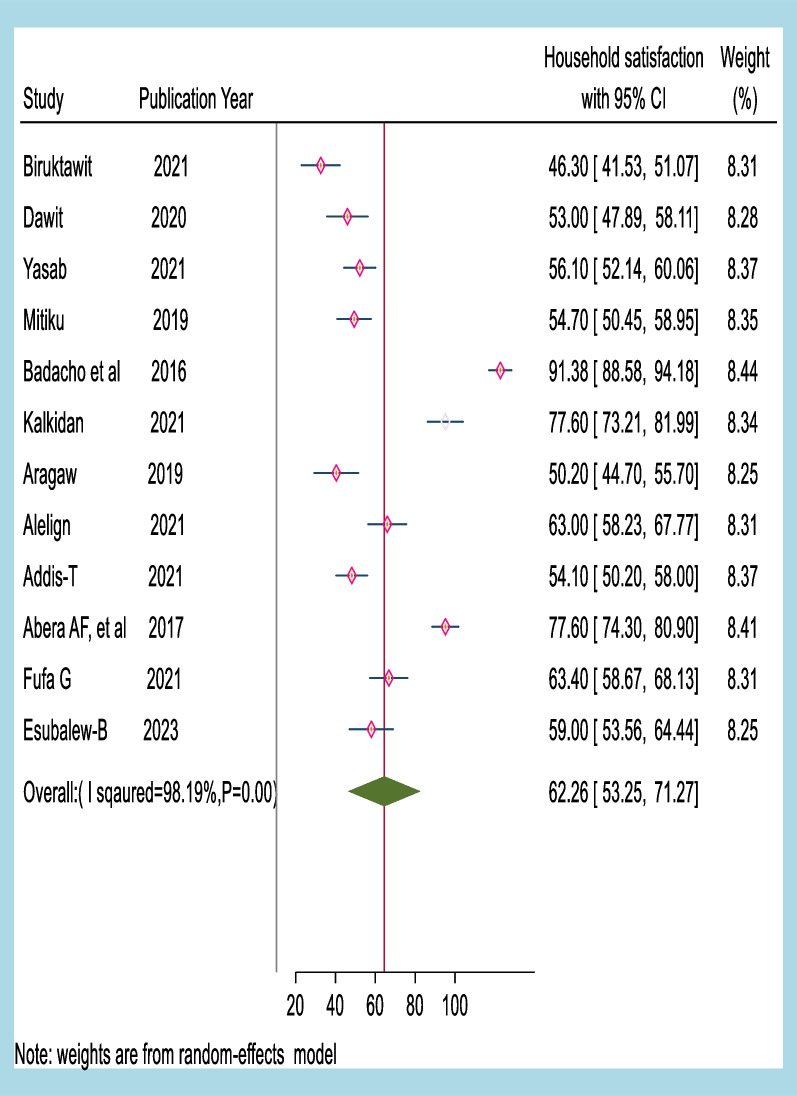
A forest plot illustrating the pooled level of household satisfaction with CBHI in Ethiopia

The findings of this study are essential for policymakers and healthcare providers in Ethiopia as they provide insight into the success and limitations of CBHI programs. The results suggest that there is a need for targeted interventions to increase the level of household satisfaction with CBHI in regions where satisfaction is lower. Additionally, the study highlights the importance of continued monitoring and evaluation of CBHI programs to ensure their sustainability and effectiveness (Fig. 2).

### Results of sensitivity analysis

The study conducted an inverse variance test (I^2^ test) among primary studies and found that there was a high degree of heterogeneity between studies (I^2^ = 98.19, *p* value < 0.00). To check the stability of the estimated effect sizes and the source of heterogeneity, the researchers used a "leave-one-out" evaluation approach as a sensitivity analysis. In this approach, they removed one study at a time and estimated the surrogacy measures using the remaining studies. This iterative procedure was used to investigate potential outlier studies on the overall effect size and identify them.

**Fig. 3:**
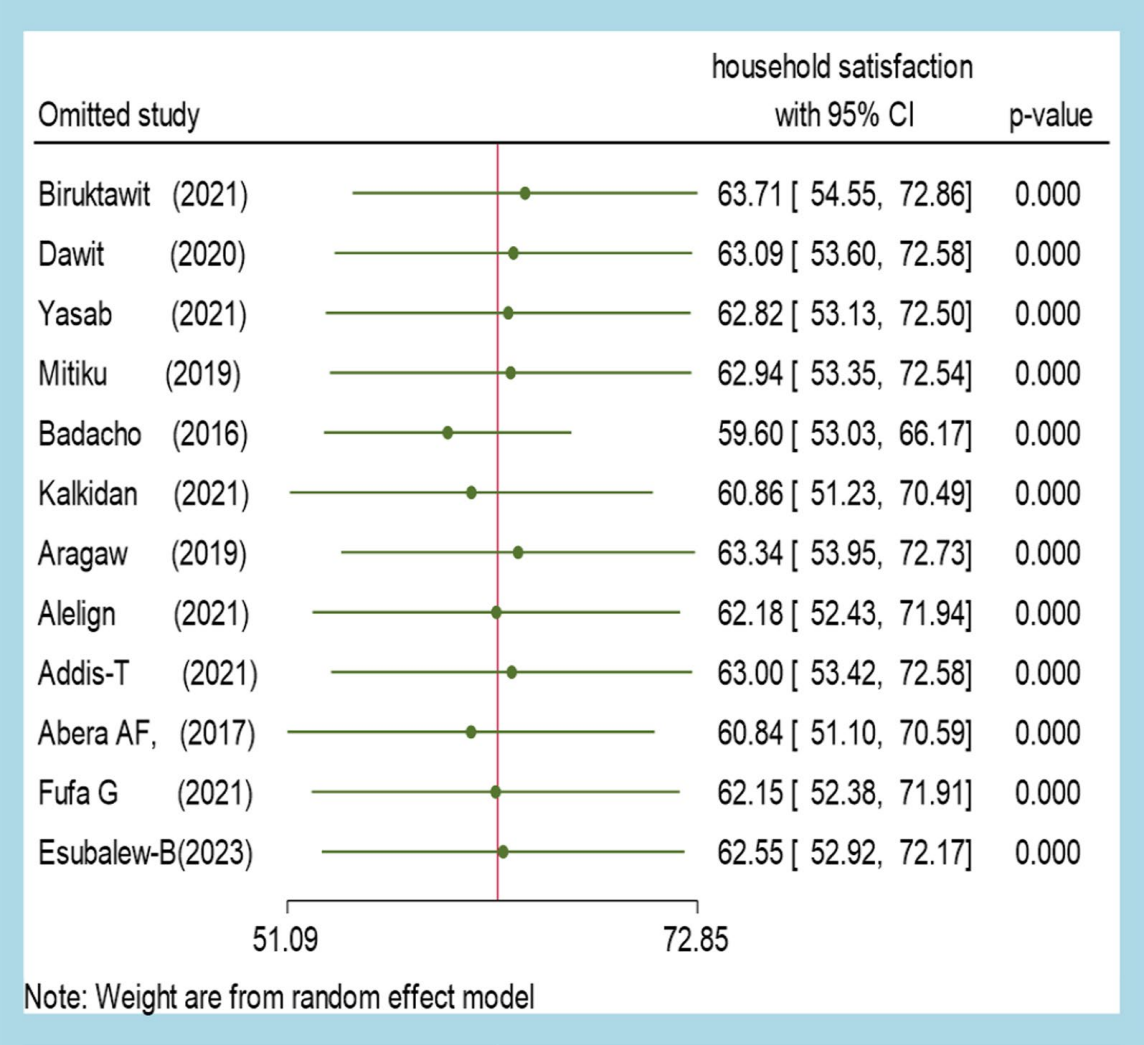
A forest plot depicting leave-one-out sensitivity analysis to estimate pooled household satisfaction with CBHI in Ethiopia

The sensitivity analysis showed that the meta-analysis finding was not dependent on a single study, and the estimated overall effect size was relatively stable. The presented effect size for each study corresponded to an overall effect size calculated from a meta-analysis excluding that study. The leave-one-out forest plot was also used to help detect outlier studies. This plot showed a vertical line at the overall effect size based on the complete set of studies (with no omission). After deleting a single study at each iteration step, the researchers found that the pooled estimate of household satisfaction varied from 59.60 (95% CL 53.03–66.17) to 63.71 (54.55–72.86) (Fig. 3). Overall, the sensitivity analysis showed that the findings of the meta-analysis were robust and not influenced by any single study.

### Subgroup analysis by region of primary study for household satisfaction with community-based health insurance in Ethiopia

We conducted a subgroup meta-analysis stratified by the primary studies' location to explore household satisfaction with CBHI. When analyzing the results based on the different regions in Ethiopia, the study found that the level of household satisfaction with CBHI was 63.40% (95% CI 58.67–68.13, I^2^ = 0.00, *p* = 0.00) in Oromia, which was the nearest to the national overall household satisfaction level. Similarly, the study found that the level of household satisfaction with CBHI was 64.01% in Amhara, which was relatively higher than in Addis Ababa city and the Oromia region.

In contrast, the study found that the level of household satisfaction with CBHI was the lowest in Addis Ababa, with a satisfaction rate of 49.68%. This is a significant finding, as Addis Ababa is the capital city of Ethiopia and has a relatively higher level of socioeconomic development compared to other regions.

Finally, the study found that the level of household satisfaction with CBHI was 66.76% (95% CI 40.12–93.40, I^2^ = 99.39, *p* = 0.06) in the South Nation Nationality and Peoples (SNNPs) region, which was the highest satisfaction rate observed in any of the regions. The high level of household satisfaction in SNNPs suggests that CBHI is a feasible and effective health financing mechanism for rural communities in Ethiopia.

**Fig. 4:**
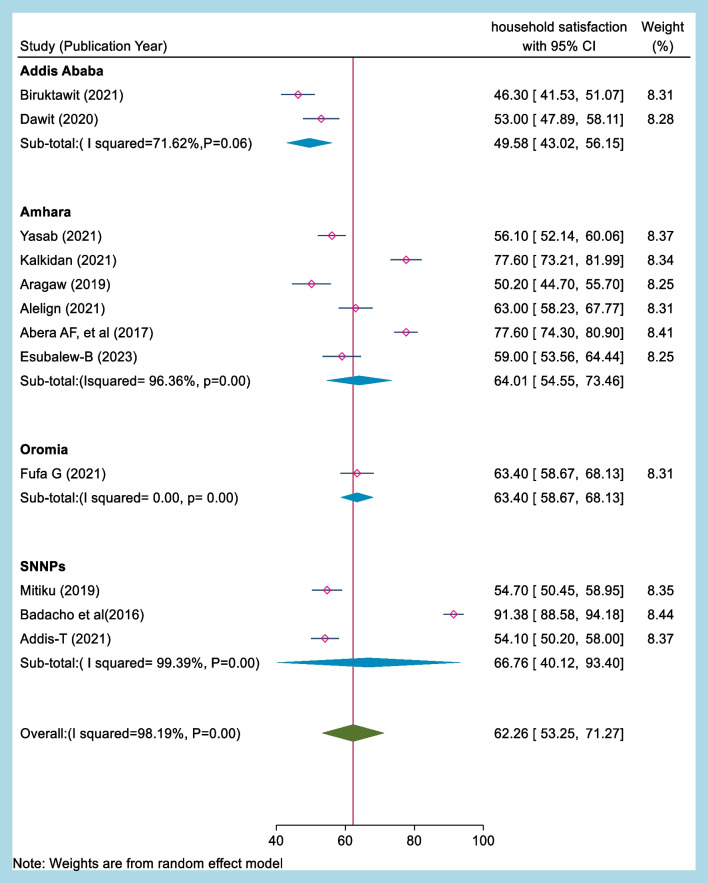
Forest plot depicting subgroup analysis by region of household satisfaction with CBHI in Ethiopia

Overall, the subgroup analysis results suggest that household satisfaction with CBHI varies across different regions in Ethiopia, with the highest satisfaction rates observed in rural regions such as SNNPs. These findings have important implications for policymakers and program implementers, as they highlight the need to tailor health financing mechanisms to the specific needs and contexts of different regions in Ethiopia (Fig. 4).

### Factors associated with household satisfaction with CBHI in Ethiopia

This systematic review and meta-analysis included 12 primary studies to investigate the association between household satisfaction and community-based health insurance (CBHI). Three out of eight studies indicated a significant association between household satisfaction and the availability of drugs within the healthcare facility providing the CBHI scheme, with a pooled odds ratio of 2.13 (95% CI 1.47–2.78). This means that households whose prescribed drugs were available in the healthcare provider were 2.13 times more likely to be satisfied than those whose prescribed medications were unavailable. Two out of three studies included to assess whether friendly services are a predictor variable showed a significant association with household satisfaction, with a pooled odds ratio of 3.85 (95% CI 1.60–6.10). The result indicated that households who got friendly services in the healthcare facility under the CBHI scheme were more likely satisfied than those who did not receive friendly services.

Two primary studies were included in a meta-analysis to assess the association between premiums and household satisfaction, and both reported that premiums were a factor associated with household satisfaction. The findings showed that households with no extra expense during healthcare visits were 2.8 times more likely to be satisfied than those who paid an additional cost from the annual premium. However, none of the three primary studies included to assess the effect of the availability of diagnostic services on household satisfaction under the CBHI scheme reported a significant association, and the pooled odds ratio from the random effect model showed an insignificant association with the outcome variable (OR 1.99; 95% CI 0.27–3.70). Furthermore, two studies analyzed qualitative data to identify factors associated with household satisfaction and CBHI. The finding supported that the availability of drugs in the healthcare facilities under the CBHI scheme and friendly services are positively associated factors with household satisfaction and community-based health insurance in Ethiopia.

Lastly, the knowledge and awareness of household heads about the CBHI scheme were associated with household satisfaction. The finding indicated that households with knowledge and awareness about CBHI were 2.52 times more likely to be satisfied than those without knowledge/awareness. These results are summarized in table (Table 3).

**Table 3:** A table depicting factors associated with household satisfaction and community-based health insurance in Ethiopia

Variables	No. studies	Study size	OR (95% CI)	I^2^ (%)	*p* value
Availability of drugs					
Yes	3	1650	2.13 (1.47–2.78)	0.00	0.00
No			1		
Friendly service					
Yes	2	997	3.85(1.60–6.10)	0.00	0.00
No			1		
Premium					
Extra-expense	2	1055	1		
No extra-expense			2.80 (1.97–3.63)	34.23	0.22
Availability of diagnostic services					
Yes	3	1046	1.99(0.27–3.70)	0.00	0.00
No			1		
Knowledge/awareness of CBHI					
Yes	2	1155	2.52 (1.73–3.30)	0.00	0.34
No			1		

## Discussion

Over the past 10 years, Ethiopia has made considerable efforts to extend CBHI coverage to every district, achieving notable progress in expanding its reach [24]. However, the enrollment rate in the community to the CBHI scheme remains low, and there is a significant dropout rate among those who do join. Unfortunately, no documented evidence at the national level explains the reasons behind this phenomenon. Therefore, this systematic review and meta-analysis study aimed to comprehensively investigate household satisfaction with community-based health insurance (CBHI) schemes in Ethiopia and its associated factors. The primary studies conducted across all regions of Ethiopia have been compiled to offer comprehensive evidence on this matter.

The random model meta-analysis found that the overall household satisfaction rate with the CBHI scheme in Ethiopia is 62.26%, suggesting a considerable level of satisfaction among households. The forest plot visualization also demonstrated regional disparities in household satisfaction with CBHI across various regions. However, it should be noted that due to the lack of systematic review and meta-analysis studies on household satisfaction with CBHI anywhere in the world, comparing this finding with individual studies conducted in other countries is not appropriate. The subgroup meta-analysis also highlighted the regional differences in household satisfaction with CBHI, with the highest satisfaction rates observed in rural regions like SNNPs. However, the lowest satisfaction rate was observed in Addis Ababa, Ethiopia's capital city, which has a higher level of socioeconomic development than other regions.

The discrepancy in satisfaction rates among regions could be due to various factors, including the limited number of studies and publications, differences in study size among studies, health-seeking behaviors, and patient flow among healthcare facilities under CBHI schemes. For example, the low household satisfaction in Addis Ababa could be due to the high patient flow in healthcare facilities in the densely populated city, which could directly impact household satisfaction with CBHI scheme healthcare services.

The findings suggest that various factors are associated with household satisfaction, including the availability of drugs, friendly services, premiums, and knowledge/awareness about the CBHI scheme. However, the availability of diagnostic services was not significantly associated with household satisfaction.

The results revealed that households whose prescribed drugs were available in the healthcare provider were more likely to be satisfied than those whose prescribed medications were not available. This finding is consistent with previous systematic review and meta-analysis studies among low-income and middle-income countries [46] that have suggested that drug availability is a critical factor influencing household satisfaction with healthcare services. The availability of drugs is an essential aspect of healthcare delivery and is associated with the quality of care patients receive. Therefore, policymakers and healthcare providers should ensure the availability of drugs to improve household satisfaction with CBHI schemes.

Moreover, the present study found that households receiving friendly services in healthcare facilities under the CBHI scheme were more likely to be satisfied than those who did not receive friendly services during healthcare visits. The finding is supported by a systematic review of studies that assessed barriers and facilitators of community-based health insurance policy renewal in low and middle-income countries [21]. This finding highlights the importance of healthcare providers' attitudes and behavior toward patients in influencing household satisfaction with healthcare services. Therefore, healthcare providers should prioritize friendly and compassionate services to improve household satisfaction with CBHI schemes.

The research findings also strongly correlate household satisfaction and healthcare costs. It was observed that households who did not incur any extra expenses during their medical visits were more likely to report satisfaction than those who had to pay additional fees from their annual premiums. Moreover, a systematic review and meta-analysis investigating the factors influencing the voluntary adoption of community-based health insurance schemes in low- and middle-income countries also recognized the significance of affordable premiums [9]. The finding is also supported by a study conducted in Ethiopia, which assessed the level and determinants of enrollment in the country's Community-Based Health Insurance (CBHI) scheme toward universal health coverage [47]. This discovery emphasizes the crucial role of affordability and financial protection in shaping household satisfaction with healthcare services. To address this issue, policymakers and healthcare providers should explore strategies to alleviate the financial burden on households, such as reducing premiums or offering subsidies.

Lastly, the research uncovered a significant association between knowledge and awareness of the CBHI scheme and household satisfaction. This outcome underscores the crucial role of education and information dissemination in enhancing household satisfaction with CBHI schemes. Policymakers and healthcare providers must prioritize initiatives to educate households about the advantages of CBHI schemes and increase awareness, ultimately improving overall household satisfaction. A systematic review and meta-analysis on the barriers and facilitators of implementing essential health packages within primary healthcare settings have supported these findings [21].

Furthermore, two studies analyzed qualitative data to identify factors associated with household satisfaction and CBHI in Ethiopia. The finding supported that the availability of drugs in healthcare facilities under the CBHI scheme and friendly services are positively associated with household satisfaction and CBHI. This section will discuss the significance of these factors and their implications for CBHI in Ethiopia.

Drug availability plays a crucial role in healthcare service provision. In Ethiopia, the healthcare system grapples with obstacles related to the accessibility and availability of drugs, which can have negative consequences on patients' health outcomes and overall satisfaction. The discovery that drug availability has a positive correlation with household satisfaction and CBHI emphasizes the significance of ensuring sufficient drug supplies in healthcare facilities operating under CBHI. This finding aligns with the conclusions drawn from a systematic review conducted on studies in low-and middle-income countries [9, 21, 46, 48].

The availability of drugs can also contribute to the success of CBHI by increasing healthcare utilization. Patients with access to affordable drugs are more likely to seek medical care and comply with treatment regimens. This, in turn, can improve health outcomes and reduce the financial burden of healthcare on households. Therefore, ensuring drug availability in healthcare facilities under CBHI is crucial for improving healthcare access, utilization, and household satisfaction.

Friendly services are another factor positively associated with household satisfaction and CBHI. Friendly services refer to the quality of care patients receive from healthcare providers, including their interpersonal skills, communication, and responsiveness to patient needs. In Ethiopia, healthcare providers often lack adequate training in patient-centered care, leading to low satisfaction and distrust among patients.

The study's findings suggest the importance of continued monitoring and evaluation of CBHI programs to ensure their sustainability and effectiveness. As such, policymakers and healthcare providers in Ethiopia should consider incorporating regular monitoring and evaluation mechanisms into CBHI programs to identify areas for improvement and enhance household satisfaction.

Based on these findings, policymakers and stakeholders can develop targeted interventions to improve household satisfaction with CBHI schemes in Ethiopia. Overall, this study contributes to the body of knowledge on healthcare financing and healthcare system improvements in Ethiopia.

The study has some limitations that should be considered when interpreting the results. For instance, the study's data sources were limited to four regions of the country; some regions did not yet have any research on household satisfaction and CBHI, which may have resulted in some bias. Additionally, the study's sample size was relatively small, which may limit the generalizability of the findings to the entire Ethiopian population. Finally, we were not able to find systematic reviews and meta-analyses elsewhere in the world, which limits our abilities to compare the level of household satisfaction with CBHI and other similar studies.

## Conclusions

The study's findings provide valuable insights into household satisfaction with CBHI in Ethiopia. The study highlights regional variations in household satisfaction and underscores the need for tailored interventions and monitoring and evaluation mechanisms to enhance CBHI programs' sustainability and effectiveness. The present study also provides valuable insights into the factors associated with household satisfaction with CBHI schemes. The findings suggest that healthcare providers and policymakers should prioritize the availability of drugs, friendly services, premium affordability, and education to improve household satisfaction with CBHI schemes. The study's findings have significant implications for policymakers and healthcare providers in Ethiopia. It is clear that regions exhibiting lower satisfaction levels require tailored interventions to enhance household contentment with CBHI. It is also essential to tailor health financing mechanisms to the specific needs and contexts of different regions in Ethiopia, as highlighted by the regional variations in household satisfaction. Future research should focus on investigating the impact of these factors on other health outcomes and exploring additional factors that influence household satisfaction with healthcare services.

## Data Availability

Data can be accessed upon reasonable request to the corresponding author.

## Abbreviations

CBHI: Community-based health insurance
OOP: Out-of-pocket
UHC: Universal health coverage
LMIC: Lower-and middle-income country
SNNPs: South Nation Nationalities and Peoples
PRISMA: Preferred reporting items for systematic review and meta-analysis
JBI: Joanna Briggs Institute
CI: Confidence interval
OR: Odds ratio
